# Performance Evaluation of Hospitals under Data Uncertainty: An Uncertain Common-Weights Data Envelopment Analysis

**DOI:** 10.3390/healthcare12060611

**Published:** 2024-03-07

**Authors:** Pejman Peykani, Mir Saman Pishvaee

**Affiliations:** School of Industrial Engineering, Iran University of Science and Technology, Tehran 1684613114, Iran

**Keywords:** hospital performance evaluation, healthcare systems, data envelopment analysis, common set of weights technique, uncertain environment, uncertainty theory

## Abstract

In the context of healthcare systems, the performance evaluation of hospitals plays a crucial role in assessing the quality of healthcare systems and facilitating informed decision-making processes. However, the presence of data uncertainty poses significant challenges to accurate performance measurement. This paper presents a novel uncertain common-weights data envelopment analysis (UCWDEA) approach for evaluating the performance of hospitals under uncertain environments. The proposed UCWDEA approach addresses the limitations of traditional data envelopment analysis (DEA) models by incorporating the uncertainty theory (UT) to model the inherent uncertainty in input and output data. Also, by utilizing a common set of weights (CSW) technique, the UCWDEA method provides a more robust and reliable assessment of hospital performance. The main advantages of the proposed UCWDEA approach can be succinctly summarized as follows. Firstly, it allows for the comparison of all hospitals on a consistent basis to calculate a realistic efficiency score, rather than an overly optimistic efficiency score. Secondly, the uncertain common-weights DEA approach exhibits linearity, enhancing its applicability. Thirdly, it possesses the capability to extend its utility under various other prevalent uncertainty distributions. Moreover, it enhances the discriminatory power of results, facilitates the ranking of hospitals in the presence of data uncertainty, and aids in identifying the sensitivity and stability levels of hospitals towards data uncertainty. Notably, in order to showcase the pragmatic application and efficacy of the uncertain common-weights DEA model, a genuine dataset has been utilized to evaluate the efficiency of 20 public hospitals in Tehran, all of which are affiliated with the Iran University of Medical Sciences. The results of the experiment demonstrate the efficacy of the UCWDEA approach in assessing and ranking hospitals amidst uncertain conditions. In summary, the research outcomes can offer policymakers valuable insights regarding hospital performance amidst data uncertainty. Additionally, it can provide practical recommendations on optimizing resource allocation, benchmarking performance, and formulating effective policies to augment the overall efficiency and effectiveness of healthcare services.

## 1. Introduction

Hospitals play a critical and central role in the healthcare system, serving as essential facilities for diagnosis, treatment, and care of patients. They are the backbone of healthcare delivery, providing a wide range of medical services, specialized treatments, and emergency care [1,2,3,4,5,6]. Consequently, their performance has a direct impact on the quality of healthcare services provided. This significance was particularly evident during global health crises, such as the coronavirus pandemic, where the level of hospital performance had a notable influence on patient mortality rates [7,8,9,10,11]. Therefore, it becomes imperative to develop an effective method for evaluating the performance and productivity of hospitals, which is considered a critical topic in the field of healthcare literature.

Assessing hospital performance is essential for several reasons. Firstly, it allows healthcare administrators and policymakers to identify areas of improvement and allocate resources effectively. By evaluating performance, hospitals can identify strengths and weaknesses in their operations, leading to targeted interventions and enhanced patient care. Secondly, performance evaluation enables benchmarking against other hospitals or industry standards, facilitating a comparative analysis that can drive improvements in healthcare delivery. Additionally, it provides a basis for accountability and transparency, as hospitals are expected to meet certain performance standards to ensure the provision of high-quality care. In general, by creating effective assessment frameworks, healthcare professionals and policymakers can gain valuable insights into the strengths and weaknesses of individual hospitals, enabling them to make informed decisions regarding resource allocation, quality improvement initiatives, and patient care optimization [12,13,14,15,16,17,18,19,20].

To address this need, researchers have proposed various methods for evaluating hospital performance. Data envelopment analysis (DEA) is a widely utilized, non-parametric, mathematical programming approach that has gained popularity among researchers in the healthcare sector [21,22,23,24,25,26]. Its primary purpose is to evaluate the efficiency and effectiveness of hospitals and their respective departments. The review highlights the benefits of using DEA as a performance measurement tool in hospitals. DEA allows for a holistic evaluation of hospital performance by considering multiple dimensions, such as patient outcomes, resource utilization, and quality of care. It supports the identification of best practices and benchmarking, facilitating performance improvement and strategic decision making within healthcare organizations. In other words, by considering multiple input and output variables, DEA enables the identification of inefficient hospitals and the assessment of performance improvement strategies.

It is important to note that DEA is commonly employed in the domains of operations research and management science (OR/MS) [27,28,29,30,31,32,33,34,35]. Its primary objective is to evaluate the relative efficiency of similar decision-making units (DMUs) by assessing their capacity to maximize output while minimizing input utilization. Notably, this approach does not require a specific functional form or make assumptions about the underlying production process, making it a flexible and adaptable method for evaluating efficiency. Furthermore, DEA provides valuable insights into the sources of inefficiency by identifying benchmark DMUs that exhibit superior performance within the dataset. By doing so, it allows for a deeper understanding of the factors contributing to inefficiencies and offers the opportunity to implement targeted improvements to enhance overall performance and productivity within the evaluated DMUs. This contributes to the continued refinement and enhancement of operations and management practices within various real-world applications, ultimately leading to improved organizational effectiveness and competitiveness.

The goal of the current research study is to introduce a new and innovative approach called uncertain common-weights data envelopment analysis (UCWDEA) to assess the performance of hospitals in a context of uncertainty. It should be explained that the proposed UCWDEA method overcomes the limitations of traditional DEA models by incorporating the principles of uncertainty theory (UT) to accurately capture the inherent uncertainty in input and output data. Moreover, by employing a common set of weights (CSW) method, the UCWDEA approach ensures a more robust and dependable assessment of hospital performance. Finally, in order to showcase the real-world applicability and efficacy of the uncertain common-weights DEA model, an authentic dataset is utilized to evaluate the efficiency of 20 Iranian public hospitals located in Tehran.

The remainder of this paper is structured as follows. In Section 2, a comprehensive review of the existing literature and identified gaps will be presented. Section 3 will provide an in-depth explanation of the uncertainty theory concept. Our novel approach, called the uncertain common-weights data envelopment analysis, will be introduced in Section 4. This approach aims to effectively evaluate the performance of hospitals when faced with uncertain data. Furthermore, in Section 5, we will apply the UCWDEA approach to a real-world case study and meticulously analyze the experimental outcomes. Lastly, Section 6 will encompass the presentation of conclusions, along with suggestions and directions for future research endeavors.

## 2. Literature Review

In this section, the literature review of DEA applications in healthcare under uncertain environments, as well as the research gaps in the literature, are presented. Accordingly, Table 1 outlines the distinctive attributes of studies incorporating the DEA model and the uncertain programming (UP) approach. It should be noted that UP approaches include stochastic optimization (SO), fuzzy optimization (FO), robust optimization (RO), bootstrap (BS), interval programming (IP), neutrosophic theory (NT), Z-number theory (ZT), and uncertainty theory (UT).

**Figure 1:**
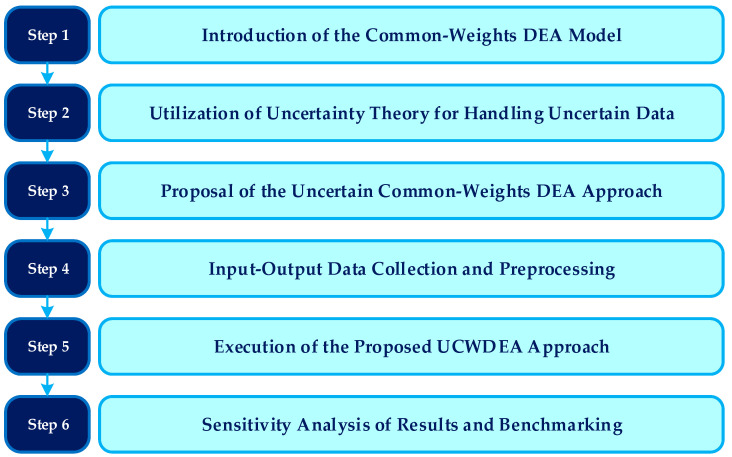
The schematic overview of sequential steps within the proposed UCWDEA approach.

As indicated in Table 1, the assessment of hospital performance in uncertain conditions has thus far neglected the integration of uncertainty theory and a common set of weights DEA model. Therefore, as demonstrated in the last row of Table 1, this study seeks to fill this research gap by proposing the UCWDEA methodology. In essence, this research paper presents a groundbreaking advancement by utilizing uncertainty theory to evaluate the performance of hospitals and the healthcare sector. It is noteworthy that a multitude of articles on uncertain DEA applications in healthcare have been published in prestigious journals, such as *Health Care Management Science* (three papers), *Expert Systems with Applications* (three papers), *Knowledge-Based Systems* (three papers), *Healthcare* (two papers), *European Journal of Operational Research* (two papers), *International Journal of Uncertainty, Fuzziness and Knowledge-Based Systems* (two papers), *Computers & Industrial Engineering* (one paper), *Soft Computing* (one paper), *Symmetry* (one paper), *Kybernetes* (one paper), and *International Journal of Fuzzy Systems* (one paper). Hence, the objective of our paper is to formulate a novel approach for uncertain data envelopment analysis and apply it to the assessment of health and performance in hospitals, marking the first instance of such utilization. By doing so, we aim to pave the way for future research endeavors in this domain, thus opening up new avenues for exploration. The research methodology of the paper is prominently depicted in Figure 1.

## 3. Uncertainty Theory

Uncertainty is an inherent characteristic of many real-world scenarios, ranging from business management and financial analysis to medical diagnosis and environmental forecasting [76,77,78,79,80]. In these domains, decision makers (DMs) often face ambiguous, incomplete, or contradictory information, which poses significant challenges when attempting to draw accurate conclusions or make informed decisions. This is where uncertainty modeling techniques, such as uncertainty theory, play a vital role. Uncertainty theory is a mathematical framework that deals with the modeling and quantification of uncertainty in various domains [81,82,83,84,85,86]. It provides concepts, methods, and techniques to represent, analyze, and reason with uncertain information. Unlike traditional probability theory, which focuses on well-defined probabilities, uncertainty theory encompasses a broader notion of uncertainty, including imprecision, vagueness, incomplete information, and subjective opinions. It allows decision makers to handle situations where data may be scarce, ambiguous, or conflicting. UT incorporates subjective beliefs, expert judgments, and subjective probabilities to capture and represent uncertain knowledge. By utilizing uncertainty theory, decision makers can make more informed decisions, assess risks more accurately, and understand the limitations of available information, leading to improved problem solving and decision making in complex and uncertain scenarios.

In uncertain theory, the belief degree function serves as a powerful tool for modeling and quantifying uncertainty in various situations [87,88,89]. In particular, it proves to be beneficial when there is a lack of sufficient data or when the available data are deemed unreliable or conflicting. By utilizing the belief degree function, it becomes possible to incorporate expert opinions and subjective judgments into the analysis and decision-making processes, fostering a more comprehensive understanding of the underlying uncertainty. The belief degree function encompasses a range of different methods and approaches that aim to capture subjective beliefs and expert opinions regarding uncertain events or variables. Uncertain theory enables decision makers to assess and quantify uncertainty in situations where traditional probability theory may not be suitable or applicable. Unlike traditional probability theory, which requires precise and complete probabilistic information, belief functions can handle imprecise, incomplete, or conflicting data. This makes them particularly useful in situations where data collection may be challenging or where the available data sources yield inconsistent or uncertain results. Rather than relying solely on statistical data, belief functions allow decision makers to incorporate a broader range of information, including expert knowledge, experience, and subjective judgments.

The belief degree function can be seen as a mathematical framework that assigns degrees of belief or uncertainty to different propositions or hypotheses. It captures the subjective opinions of experts or decision makers, allowing them to express their confidence or lack thereof in specific assertions. These beliefs are represented using belief degrees, which convey the level of support or disbelief in a particular proposition. The belief degrees are characterized by a set of mathematical rules that govern their manipulation and interpretation. One of the key advantages of the belief degree function is its ability to handle conflicting information. In real-world scenarios, experts may hold divergent opinions or have access to different pieces of evidence, leading to conflicting conclusions. The belief degree function provides a way to aggregate and combine these diverse viewpoints, resulting in a comprehensive representation of uncertainty that considers all available information. For example, in the field of medical diagnosis, the belief degree function allows healthcare professionals to evaluate the uncertainty associated with different diagnostic hypotheses, taking into account the opinions and expertise of multiple specialists.

Now, consider a situation where † represents a set that is not empty, and ℧ denotes a collection of subsets of †. Additionally, ℧ is established as a σ-algebra over †. The pair (? ℧) is commonly known as a measurable space, and any member ∇ within this measurable space is referred to as an event. An uncertain measure is denoted as function Θ:℧→[0,1], and it satisfies four axioms, namely normality, duality, subadditivity, and product [90,91,92]. Consequently, the triplet (? ℧,Θ) is defined as an uncertainty space. For an uncertain variable ϖ, the uncertainty distribution Φ is defined as Equation (1):

(1) $$\Phi (\tau )=\Psi \left\{\varpi \leq \tau \right\},\tau \in ℜ$$

It should be explained that a regular uncertainty distribution Φ refers to the distribution of an uncertain variable ϖ, where the inverse function $\Phi^{-1}(\xi )$ exists uniquely for every value ξ∈[0,1]. In such instances, the inverse function $\Phi^{-1}(\xi )$ is known as the inverse uncertainty distribution of ϖ.

Notably, one prominent concept within uncertainty theory is the linear uncertainty distribution, which is used to model uncertainty based on a linear relationship between the lower and upper bounds of a given variable. This distribution provides a straightforward and intuitive representation of uncertainty, particularly in situations where the possible range of values can be described in a linear fashion. The linear uncertainty distribution ℒ$\left(\underline{\alpha },\bar{\alpha }\right)$ is defined as Equation (2), in which $\underline{\alpha }$ and $\bar{\alpha }$ are real numbers with $\underline{\alpha }<\bar{\alpha }$:

(2) $$\Phi (\tau )=\left\{\begin{array}{ll} 0, & if\;\tau \leq \underline{\alpha };\\ \frac{\left(\tau -\underline{\alpha }\right)}{\left(\bar{\alpha }-\underline{\alpha }\right)}, & if\;\underline{\alpha }\leq \tau \leq \bar{\alpha };\\ 1, & if\;\bar{\alpha }\leq \tau . \end{array}\right.$$

Also, the inverse uncertainty distribution of ℒ$\left(\underline{\alpha },\bar{\alpha }\right)$ is as Equation (3):

(3) $$\Phi^{-1}(\xi )=(1-\xi )\underline{\alpha }+(\xi )\bar{\alpha }$$

Moreover, the zigzag uncertainty distribution offers a different approach to characterizing uncertainty. This distribution is defined by a series of linear segments that form a zigzag pattern, reflecting the varying degrees of uncertainty across different intervals or regions. The zigzag uncertainty distribution is particularly useful for capturing irregular or non-linear uncertainty patterns and has applications in various fields, such as risk assessment and decision analysis. The zigzag uncertainty distribution Ƶ$\left(\underline{\beta },\beta ,\bar{\beta }\right)$ is defined as Equation (4), in which $\underline{\beta }$, β, and $\bar{\beta }$ are real numbers with $\underline{\beta }<\beta <\bar{\beta }$:

(4) $$\Phi (\tau )=\left\{\begin{array}{ll} 0, & if\;\tau \leq \underline{\beta };\\ \frac{\left(\tau -\underline{\beta }\right)}{2(\beta -\underline{\beta })}, & if\;\underline{\beta }\leq \tau \leq \beta ;\\ \frac{\left(\tau +\bar{\beta }-2\beta \right)}{2(\bar{\beta }-\beta )}, & if\;\beta \leq \tau \leq \bar{\beta };\\ 1, & if\;\bar{\beta }\leq \tau . \end{array}\right.$$

Also, the inverse uncertainty distribution Ƶ$\left(\underline{\beta },\beta ,\bar{\beta }\right)$ is as Equation (5):

(5) $$\Phi^{-1}(\xi )=\left\{\begin{array}{ll} (1-2\xi )\underline{\beta }+(2\xi )\beta , & if\;\xi \leq 0.5;\\ (2-2\xi )\beta +(2\xi -1)\bar{\beta }, & if\;\xi >0.5. \end{array}\right.$$

Finally, in the context of uncertainty theory, the normal uncertainty distribution is employed to represent the uncertainty or variability associated with a parameter or variable. It is characterized by a bell-shaped curve and is widely used to model the probability distribution of a continuous random variable. In this distribution, the mean, which represents the central or most likely value, and the standard deviation, which measures the spread or variability of the variable around the mean, are the key parameters that define the shape of the distribution. The normal uncertainty distribution is widely utilized in various fields, such as finance, engineering, and risk management, owing to its mathematical tractability and the prevalence of phenomena that naturally exhibit a bell-shaped distribution. Its application provides a valuable means of capturing uncertainty and risk, ultimately supporting robust decision making and planning under conditions of variability and unknown outcomes. The normal uncertainty distribution ℕ(μ,δ) is defined as Equation (6), in which μ and σ are real numbers with σ>0:

(6) $$\Phi (\tau )={\left(1+exp\left(\frac{\pi (\mu -\tau )}{\sqrt{3}\sigma }\right)\right)}^{-1},\tau \in ℜ$$

Also, the inverse uncertainty distribution ℕ(μ,δ) is as Equation (7):

(7) $$\Phi^{-1}(\xi )=\mu +\frac{\sqrt{3}\sigma }{\pi }ln\left(\frac{\xi }{1-\xi }\right)$$

It is important to highlight that uncertainty theory and uncertain measures play a crucial role in capturing and representing the deterministic equivalent of uncertain chance constraints within mathematical optimization problems. In scenarios where decision making involves optimization under uncertainty, particularly when considering risk or stochastic variability, uncertain chance constraints become significant in ensuring robust and reliable solutions. By leveraging uncertainty theory, which provides a framework for reasoning about imprecise or ambiguous information, and by utilizing uncertain measures to quantify the extent of uncertainty associated with specific variables or constraints, practitioners can effectively translate uncertain chance constraints into deterministic equivalents. This process involves reformulating the original optimization problem to account for the potential variability or ambiguity in relevant parameters, ultimately yielding a deterministic problem that addresses the probabilistic nature of the original constraints.

Employing uncertain measures allows decision makers to capture and incorporate the inherent uncertainty in optimization problems, thus providing a means to assess and mitigate risks while striving to achieve desirable outcomes. The integration of uncertainty theory and uncertain measures into optimization models enables decision makers to make informed, robust decisions by considering a spectrum of potential outcomes and associated probabilities, leading to more resilient and adaptive solutions in the face of uncertainty. In general, the application of uncertainty theory and uncertain measures to represent the deterministic equivalent of uncertain chance constraints in mathematical optimization problems underscores their significance in addressing real-world decision-making scenarios characterized by ambiguity, variability, and risk.

## 4. The Proposed UCWDEA Approach

In traditional DEA, each decision-making unit is evaluated individually, and unique weights are assigned to each DMU based on its specific characteristics. However, in the common-weights technique, a single set of weights is used to evaluate all DMUs in the analysis. This approach allows for a more standardized and consistent evaluation across different DMUs as it eliminates the potential bias that may arise from using DMU-specific weights. By using a common set of weights, the technique aims to provide a fair and objective comparison of efficiency among decision-making units [93,94,95,96,97]. The common-weights technique in DEA can be particularly useful when comparing DMUs within the same industry or sector, as it allows for a more direct comparison of their relative efficiency. It helps to identify best practices and areas for improvement by highlighting DMUs that are operating at maximum efficiency and those that have room for improvement. Overall, the common-weights technique in data envelopment analysis provides a standardized and objective approach to evaluate the efficiency of decision-making units by using a common set of weights for all DMUs in the analysis.

Let us consider a situation where we have a group of S similar decision-making units $DMU_{g}(g=1,2,\ldots ,S)$. These DMUs are responsible for transforming F inputs, denoted as $p_{ig}(i=1,2,\ldots ,F)$, into H outputs, denoted as $q_{jg}(j=1,2,\ldots ,H)$. Additionally, we assign non-negative weights $v_{i}(i=1,2,\ldots ,F)$ to the inputs and $u_{j}(j=1,2,\ldots ,H)$ to the outputs. Based on the CSW technique, the efficiency score of all DMUs can be determined by solving the subsequent multi-objective programming (MOP) problem:

(8) $$Max\;\;\;\;\Psi_{g}=\frac{\sum_{j=1}^{H}q_{jg}u_{j}}{\sum_{i=1}^{F}p_{ig}v_{i}},\;\;\;\;\forall g$$

$$S.t.\;\;\;\;\Psi_{g}=\frac{\sum_{j=1}^{H}q_{jg}u_{j}}{\sum_{i=1}^{F}p_{ig}v_{i}}\leq 1,\;\;\;\;\forall g$$

$$v_{i},u_{j}\geq \varepsilon ,\;\;\;\;\forall i,j$$

By employing the goal programming (GP) technique and utilizing the deviational variables of $k_{g}^{+}$ and $k_{g}^{-}$, we can reformulate Model (8) as Model (9):

(9) $$Min\;\;\;\;\sum_{g=1}^{S}\left(k_{g}^{+}+k_{g}^{-}\right)$$

$$S.t.\;\;\;\;\frac{\sum_{j=1}^{H}q_{jg}u_{j}+k_{g}^{+}}{\sum_{i=1}^{F}p_{ig}v_{i}-k_{g}^{-}}=1,\;\;\;\forall g$$

$$k_{g}^{+},k_{g}^{-}\geq 0,\;\;\;\forall g$$

$$v_{i},u_{j}\geq \varepsilon ,\;\;\;\forall i,j$$

Moreover, the linear representation of Model (9) is introduced as Model (10), incorporating the variable $k_{g}=k_{g}^{+}+k_{g}^{-}$.

(10) $$Min\;\;\;\;\sum_{g=1}^{S}k_{g}$$

$$S.t.\;\;\;\;\sum_{j=1}^{H}q_{jg}u_{j}-\sum_{i=1}^{F}p_{ig}v_{i}+k_{g}=0,\;\;\;\;\forall g$$

$$k_{g}\geq 0,\;\;\;\;\forall g$$

$$v_{i},u_{j}\geq \varepsilon ,\;\;\;\;\forall i,j$$

In the event that $v_{i}^{*}$, $u_{j}^{*}$ and $k_{g}^{*}$ represent the optimal solutions of Model (10), the efficiency scores of the $DMU_{g}$ can be acquired utilizing Equation (11):

(11) $$\Psi_{g}^{*}=\frac{\sum_{j=1}^{H}q_{jg}u_{j}^{*}}{\sum_{i=1}^{F}p_{ig}v_{i}^{*}}=1-\frac{k_{g}^{*}}{\sum_{i=1}^{F}p_{ig}v_{i}^{*}},\;\;\;\;\forall g$$

Let us consider a situation where the inputs ${\tilde{p}}_{ig}(i=1,2,\ldots ,F)$ and outputs ${\tilde{q}}_{jg}(j=1,2,\ldots ,H)$ are characterized by uncertainty and they follow a linear, uncertain distribution, denoted as ℒ$\left({\underline{p}}_{ig},{\bar{p}}_{ig}\right)$ and ℒ$\left({\underline{q}}_{jg},{\bar{q}}_{jg}\right)$, respectively. Subsequently, by applying an uncertain measure in accordance with Equation (3), the deterministic representation of the UCWDEA model is introduced as Model (12) for the specified belief degree ξ:

(12) $$Min\;\;\;\;\sum_{g=1}^{S}k_{g}$$

$$S.t.\;\;\;\;\sum_{j=1}^{H}\left((1-\xi ){\underline{q}}_{jg}+(\xi ){\bar{q}}_{jg}\right)u_{j}-\sum_{i=1}^{F}\left((\xi ){\underline{p}}_{ig}+(1-\xi ){\bar{p}}_{ig}\right)v_{i}+k_{g}=0,\;\;\;\;\forall g$$

$$k_{g}\geq 0,\;\;\;\;\forall g$$

$$v_{i},u_{j}\geq \varepsilon ,\;\;\;\;\forall i,j$$

Finally, if $v_{i}^{*(\xi )}$, $u_{j}^{*(\xi )}$ and $k_{g}^{*(\xi )}$ symbolize the optimal solutions of Model (12), then the efficiency scores of the $DMU_{g}$ can be obtained by employing Equation (13) when considering the desired belief degree.

(13) $$\Psi_{g}^{*(\xi )}=\frac{\sum_{j=1}^{H}\left((1-\xi ){\underline{q}}_{jg}+(\xi ){\bar{q}}_{jg}\right)u_{j}^{*(\xi )}}{\sum_{i=1}^{F}\left((\xi ){\underline{p}}_{ig}+(1-\xi ){\bar{p}}_{ig}\right)v_{i}^{*(\xi )}}=1-\frac{k_{g}^{*(\xi )}}{\sum_{i=1}^{F}\left((\xi ){\underline{p}}_{ig}+(1-\xi ){\bar{p}}_{ig}\right)v_{i}^{*(\xi )}},\;\;\;\;\forall g$$

In conclusion, a novel uncertain common-weights data envelopment analysis approach is introduced as Model (12), which effectively evaluates the performance of hospitals in the presence of uncertain data characterized by a linear distribution. In the subsequent section, we will delve into the practical application of the UCWDEA method using authentic data.

## 5. Case Study and Experimental Results

In this section, we aim to showcase the usefulness and efficacy of the proposed uncertain common-weights DEA approach by utilizing a genuine dataset to evaluate the performance of 20 public hospitals in Iran. Through various perspectives, the features and characteristics deemed essential for the evaluation of hospital performance may be categorized as follows:

Operational Viewpoint: number of beds, number of staff, score of equipment and facilities, score of location and infrastructures, number of outpatients, number of inpatients, and number of surgeries.

Financial Viewpoint: operational costs, revenue generation, budget allocation, profit margins, return on investment, and financial stability.

Patient Care Viewpoint: quality of care indicators, patient satisfaction scores, adherence to best practices, patient outcomes, patient experience, and quality of care provided.

It is pertinent to clarify that this paper assesses hospital performance through an operational viewpoint. Accordingly, four inputs and three outputs are considered for performance assessment of hospitals. The four input variables include: number of beds (NOBE), number of staff (NOST), score of equipment and facilities (SOEF), and score of location and infrastructures (SOLI). Also, the three output variables include: number of outpatients (NOOU), number of inpatients (NOIN), and number of surgeries (NOSU). Below, we present the explanations regarding each of the input and output variables.

Number of Beds: The number of beds is a crucial metric for evaluating a hospital’s capacity and service delivery. It reflects the hospital’s ability to accommodate patients and provide inpatient care services. A hospital with a larger number of beds may be better equipped to handle varying patient loads, cater to a wider range of medical needs, and potentially reduce wait times for admissions.

Number of Staff: The number of staff members, including medical, nursing, administrative, and support personnel, is a significant factor in assessing a hospital’s performance. Adequate staffing levels contribute to the efficient delivery of care, patient safety, and overall operational effectiveness. Staffing ratios and skill mix are also important considerations, as they impact the quality of patient care and the workload distribution among healthcare professionals.

Score of Equipment and Facilities: The quality and adequacy of medical equipment and facilities directly influence a hospital's ability to diagnose, treat, and care for patients. Evaluating the maintenance, availability, and technological sophistication of equipment, such as imaging machines, surgical tools, and patient monitoring systems, is essential. Additionally, assessing the condition and functionality of facilities, including patient rooms, operating theaters, and support areas, informs the overall capability of the hospital to deliver high-quality care.

Score of Location and Infrastructures: The hospital’s location and infrastructural attributes, such as accessibility, transportation connectivity, and safety considerations, play a significant role in its performance evaluation. Proximity to population centers, public transportation, and other healthcare facilities can impact patient access to services. Adequate parking, safety measures, and the overall condition and design of the physical infrastructure also contribute to the hospital’s appeal and convenience for patients, visitors, and staff.

Number of Outpatients: The number of outpatients refers to the count of individuals who receive medical services or treatment at a hospital without being admitted as inpatients. This metric reflects the hospital’s ability to provide ambulatory care, such as consultations, diagnostic procedures, minor treatments, and follow-up appointments, to individuals who do not require a hospital stay. The volume of outpatients serves as an indicator of the hospital’s role in addressing community healthcare needs and managing non-emergent or chronic conditions.

Number of Inpatients: The number of inpatients represents the count of individuals who are admitted to the hospital for medical treatment or care that necessitate an overnight stay or longer. This metric reflects the hospital’s capacity to provide acute and specialized medical services, including inpatient surgeries, intensive care, and complex treatments, as well as long-term care for conditions requiring extended hospitalization.

Number of Surgeries: The number of surgeries performed by a hospital encompasses various types of surgical procedures, including elective, emergency, inpatient, and outpatient surgeries. This metric is indicative of the hospital’s surgical volume and specialty services and the scope of its surgical capabilities. It provides insights into the hospital’s role in managing surgical conditions and addressing healthcare needs related to specific medical specialties and its overall contribution to surgical care within the community or region it serves.

Table 2 and Table 3 demonstrate the collection of input and output variables for 20 Iranian hospitals, respectively. These variables are represented under a linear uncertainty distribution. It is crucial to specify that the research data have been compiled for 20 public hospitals located in Tehran, all of which are under the authority of the Iran University of Medical Sciences.

It should be explained that the data uncertainty observed in the hospital data presented in Table 2 and Table 3 pertains to the lack of precise or accurate information in a particular context. It could arise due to various factors, such as incomplete or outdated data sources, discrepancies in reporting, or variations in how the data are collected and recorded. For example, in a healthcare setting, the number of beds in a hospital may vary over time due to factors like renovations, temporary closures, or changes in capacity. Additionally, different sources or databases may provide conflicting information about the number of beds, leading to uncertainty. Data uncertainty in a hospital can impact decision-making processes that rely on this information. It is important to acknowledge and account for this uncertainty when analyzing or using such data to avoid potential inaccuracies or misinterpretations.

By incorporating uncertainty theory or related methods, we can better account for these uncertainties and quantify their impact on decision-making processes. It allows us to assess the reliability and robustness of the data, providing a more comprehensive understanding of the possible outcomes and associated risks. Applying uncertainty theory helps to avoid a purely deterministic perspective that assumes absolute certainty in the data. Instead, it acknowledges the inherent variability and provides a framework for making informed decisions while considering the potential range of outcomes. Overall, uncertainty theory provides a more realistic and comprehensive approach to analyzing and interpreting solid data by accounting for the uncertainties that may be present, leading to more robust decision-making processes.

The results obtained from the UCWDEA approach, including $k_{g}^{*(\xi )}$ and $\sum_{g=1}^{S}k_{g}^{*(\xi )}$ as well as $\Psi_{g}^{*(\xi )}$ under five belief degrees including 0%, 25%, 50%, 75%, and 100%, are presented in Table 4 and Table 5, respectively. It is necessary to explain that the LINGO 20 Software was utilized for solving the models.

**Figure 2:**
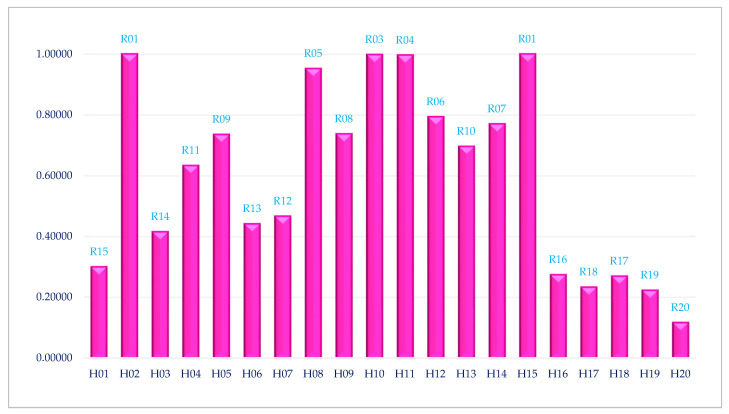
The Final Efficiency Scores of 20 Iranian Hospitals Based on the UCWDEA Approach. Abbreviations: H01–H20: Hospital 01–Hospital 20; R01–R20: Rank 01–Rank 20.

Finally, hospitals are assessed and ranked by calculating the average efficiency scores across various belief degrees. The outcomes, which determine the hospital rankings, are illustrated in Figure 2.

As can be seen in Figure 2, Hospitals 2 and 15 exhibit superior performance when compared to other hospitals, making them ideal candidates for analyzing their performance and planning. These hospitals can serve as benchmarks for other hospital managements. Generally, by employing the proposed UCWDEA approach, researchers can assess hospital performance under data uncertainty, providing a more comprehensive understanding of their efficiency and productivity. This approach considers the inherent uncertainties in healthcare data, allowing for a more accurate evaluation of hospital performance in real-world scenarios. Ultimately, the development and application of effective methods like uncertain common-weights DEA contribute to the advancement of healthcare literature and support evidence-based decision making in healthcare management. Through the utilization of the UCWDEA approach, decision makers can gain a comprehensive understanding of the efficiency and effectiveness of hospitals by considering the uncertainties present in the healthcare environment. The proposed UCWDEA approach facilitates better-informed decision-making processes and enables the identification of best practices and benchmarks in hospital performance.

The results of this study can be valuable for policymakers in the healthcare sector. Policymakers can practically apply the findings of this research in several ways. Firstly, by understanding the efficiency levels of hospitals under data uncertainty, policymakers can identify areas where resources are being underutilized or misallocated. This information can help in making informed decisions on resource allocation and improving overall healthcare service delivery. Secondly, this study can assist policymakers in benchmarking different hospitals against each other based on their performance under uncertain data conditions. By identifying high-performing hospitals, policymakers can learn from their best practices and implement strategies to enhance the efficiency of underperforming hospitals. Furthermore, the research results can guide policymakers in developing targeted interventions and policies to address specific inefficiencies or challenges faced by hospitals in uncertain data environments. By tailoring policies based on the insights gained from this study, policymakers can drive improvements in healthcare quality, accessibility, and cost-effectiveness. In summary, the findings of this research can provide policymakers with valuable insights into the performance of hospitals under data uncertainty and offer practical guidance on how to optimize resource allocation, benchmark performance, and design effective policies to enhance the overall efficiency and effectiveness of healthcare services.

## 6. Conclusions and Future Research Directions

The performance and productivity of hospitals directly impact the overall healthcare system. Efficient and effective hospitals not only provide high-quality care to patients but also contribute to the overall health outcomes of the population. Evaluating the performance of hospitals is crucial for identifying areas of improvement, optimizing resource allocation, and enhancing patient satisfaction and outcomes. Given the complexity and dynamic nature of healthcare systems, developing an effective method for evaluating hospital performance is of utmost importance. This allows policymakers, administrators, and healthcare professionals to make informed decisions, implement evidence-based practices, and allocate resources efficiently. By continuously monitoring and evaluating hospital performance, healthcare systems can strive for continuous improvement, ensuring the delivery of high-quality and patient-centered care. Accordingly, the primary objective of the present research study is to propose an inventive and advanced method known as uncertain common-weights DEA for evaluating hospital performance in contexts characterized by uncertainty.

One potential limitation of this research on the performance evaluation of hospitals under data uncertainty is the exclusion of external factors and dynamic environments. These external variables could encompass a range of elements, such as governmental policy shifts, changes in healthcare regulations, economic fluctuations, or even technological advancements within the healthcare sector. By not accounting for these external influences, the research may overlook crucial dynamics that could significantly impact the performance evaluation of hospitals. For instance, a sudden policy change favoring certain healthcare facilities could skew the results in favor of those institutions, even if their actual performance metrics remain unchanged. Similarly, economic downturns or fluctuations in patient demographics could introduce uncertainties that are not captured within the study framework.

Therefore, while this research provides valuable insights into hospital performance evaluation under data uncertainty, the absence of external factors as part of the analysis poses a limitation that could affect the overall robustness and generalizability of the findings. Future studies could address this limitation by incorporating a more comprehensive approach that considers the broader contextual factors influencing hospital performance within uncertain environments. Also, to further advance research in this field, alternative and widely used uncertain programming techniques, like uncertainty set-based robust optimization [49,98,99,100,101], scenario-based robust optimization [102,103,104,105,106], robust possibilistic programming [107,108,109,110,111], and fuzzy chance-constrained programming [112,113,114,115,116], can be incorporated to effectively address the challenges posed by data uncertainty.

## Figures and Tables

**Table 1:** Data envelopment analysis and uncertain programming in healthcare: A literature review.

Year	Research	DEA Model	Uncertain Programming Approach							
			SO	FO	RO	BS	IP	NT	ZT	UT
1999	Morita and Seiford [36]	Charnes–Cooper–Rhodes	✓							
2012	Ebrahimnejad [37]	Cost Efficiency		✓						
2012	Hatami-Marbini et al. [38]	Malmquist Productivity Index		✓						
2012	Khaki et al. [39]	Charnes–Cooper–Rhodes			✓					
2013	Costantino et al. [40]	Cross Efficiency		✓						
2013	De Nicola et al. [41]	Banker–Charnes–Cooper				✓				
2013	Khodaparasti and Maleki [42]	Simultaneous Dynamic		✓						
2014	Kalantary and Azar [43]	Charnes–Cooper–Rhodes			✓					
2014	Karadayi and Karsak [44]	Charnes–Cooper–Rhodes		✓						
2015	Dotoli et al. [45]	Cross Efficiency		✓						
2015	Haji-Sami et al. [46]	Charnes–Cooper–Rhodes			✓					
2015	Kheirollahi et al. [47]	Congestion	✓							
2015	Mitropoulos et al. [48]	Charnes–Cooper–Rhodes	✓							
2016	Rabbani et al. [49]	Charnes–Cooper–Rhodes			✓	✓	✓			
2016	Shwartz et al. [50]	Banker–Charnes–Cooper				✓				
2017	Arya and Yadav [51]	Slacks-Based Measure		✓						
2017	Karsak and Karadayi [52]	Charnes–Cooper–Rhodes		✓						
2017	Kheirollahi et al. [53]	Congestion		✓						
2017	Otay et al. [54]	Charnes–Cooper–Rhodes		✓						
2018	Ahmadvand and Pishvaee [55]	Common Set of Weights		✓						
2018	Peykani et al. [56]	Malmquist Productivity Index		✓						
2018	Wu and Wu [57]	Charnes–Cooper–Rhodes			✓					
2019	Hatefi and Haeri [58]	Charnes–Cooper–Rhodes		✓						
2019	Ji et al. [59]	Charnes–Cooper–Rhodes		✓						
2019	Peykani et al. [60]	Charnes–Cooper–Rhodes		✓						
2020	Arya and Yadav [61]	Charnes–Cooper–Rhodes		✓						
2020	Ghafari Someh et al. [62]	Three-Stage Network					✓			
2020	Yang et al. [63]	Charnes–Cooper–Rhodes						✓		
2021	Abdelfattah [64]	Charnes–Cooper–Rhodes						✓		
2021	Gómez-Gallego et al. [65]	Banker–Charnes–Cooper		✓						
2021	Izadikhah et al. [66]	Charnes–Cooper–Rhodes		✓			✓			
2021	Jahani and Kordrostami [67]	Slacks-Based Measure		✓						
2021	Tavana et al. [68]	Charnes–Cooper–Rhodes							✓	
2022	Izadikhah [69]	Slacks-Based Measure	✓	✓						
2022	Omrani et al. [70]	Banker–Charnes–Cooper		✓	✓					
2022	Peykani et al. [71]	Window Analysis		✓						
2023	Cinaroglu [72]	Charnes–Cooper–Rhodes		✓						
2023	Ferreira et al. [73]	Directional Distance	✓		✓	✓				
2023	Jahani and Kordrostami [74]	Inverse Directional Distance		✓						
2023	Mohanta et al. [75]	Charnes–Cooper–Rhodes						✓		
The Current Research		Common Set of Weights								✓

**Table 2:** The dataset for inputs of 20 Iranian public hospitals.

Hospitals	NOBE	NOST	SOEF	SOLI
Hospital 01	ℒ (114, 126)	ℒ (338, 374)	ℒ (53, 59)	ℒ (68, 76)
Hospital 02	ℒ (429, 475)	ℒ (834, 922)	ℒ (94, 100)	ℒ (90, 100)
Hospital 03	ℒ (134, 148)	ℒ (316, 350)	ℒ (57, 63)	ℒ (63, 69)
Hospital 04	ℒ (271, 299)	ℒ (379, 419)	ℒ (71, 79)	ℒ (65, 71)
Hospital 05	ℒ (116, 128)	ℒ (344, 380)	ℒ (55, 61)	ℒ (59, 65)
Hospital 06	ℒ (72, 80)	ℒ (177, 195)	ℒ (49, 55)	ℒ (43, 47)
Hospital 07	ℒ (119, 131)	ℒ (299, 331)	ℒ (45, 49)	ℒ (56, 62)
Hospital 08	ℒ (89, 99)	ℒ (166, 184)	ℒ (50, 56)	ℒ (33, 37)
Hospital 09	ℒ (85, 93)	ℒ (212, 234)	ℒ (45, 49)	ℒ (63, 69)
Hospital 10	ℒ (19, 21)	ℒ (72, 80)	ℒ (23, 25)	ℒ (33, 37)
Hospital 11	ℒ (23, 25)	ℒ (95, 105)	ℒ (32, 36)	ℒ (43, 47)
Hospital 12	ℒ (74, 82)	ℒ (201, 223)	ℒ (37, 41)	ℒ (44, 48)
Hospital 13	ℒ (29, 32)	ℒ (90, 100)	ℒ (33, 37)	ℒ (31, 35)
Hospital 14	ℒ (166, 184)	ℒ (304, 336)	ℒ (74, 82)	ℒ (55, 61)
Hospital 15	ℒ (69, 77)	ℒ (102, 112)	ℒ (35, 39)	ℒ (41, 45)
Hospital 16	ℒ (71, 79)	ℒ (166, 184)	ℒ (88, 98)	ℒ (84, 92)
Hospital 17	ℒ (111, 123)	ℒ (359, 397)	ℒ (91, 99)	ℒ (84, 92)
Hospital 18	ℒ (88, 98)	ℒ (200, 221)	ℒ (70, 78)	ℒ (80, 88)
Hospital 19	ℒ (86, 95)	ℒ (246, 272)	ℒ (89, 99)	ℒ (88, 98)
Hospital 20	ℒ (42, 46)	ℒ (93, 103)	ℒ (37, 41)	ℒ (60, 66)

Abbreviations: NOBE: number of beds; NOST: number of staff; SOEF: score of equipment and facilities; SOLI: score of location and infrastructures.

**Table 3:** The dataset for outputs of 20 Iranian public hospitals.

Hospitals	NOOU	NOIN	NOSU
Hospital 01	ℒ (49684, 54913)	ℒ (6114, 6757)	ℒ (1774, 1960)
Hospital 02	ℒ (360182, 398096)	ℒ (39230, 43360)	ℒ (18355, 20287)
Hospital 03	ℒ (64230, 70991)	ℒ (8434, 9322)	ℒ (3057, 3379)
Hospital 04	ℒ (121546, 134341)	ℒ (17598, 19451)	ℒ (2926, 3234)
Hospital 05	ℒ (118536, 131013)	ℒ (16699, 18456)	ℒ (527, 583)
Hospital 06	ℒ (41049, 45370)	ℒ (5363, 5927)	ℒ (1710, 1890)
Hospital 07	ℒ (68156, 75330)	ℒ (6209, 6862)	ℒ (4012, 4434)
Hospital 08	ℒ (85592, 94602)	ℒ (7443, 8227)	ℒ (1097, 1213)
Hospital 09	ℒ (94913, 104904)	ℒ (7882, 8711)	ℒ (1664, 1840)
Hospital 10	ℒ (54048, 59737)	ℒ (3549, 3922)	ℒ (808, 893)
Hospital 11	ℒ (65790, 72716)	ℒ (9216, 10186)	ℒ (410, 454)
Hospital 12	ℒ (83392, 92170)	ℒ (8237, 9104)	ℒ (2076, 2294)
Hospital 13	ℒ (40613, 44888)	ℒ (3273, 3618)	ℒ (599, 662)
Hospital 14	ℒ (118915, 131432)	ℒ (10838, 11979)	ℒ (5545, 6129)
Hospital 15	ℒ (74349, 82175)	ℒ (6533, 7221)	ℒ (1959, 2165)
Hospital 16	ℒ (32568, 35996)	ℒ (4308, 4761)	ℒ (3092, 3418)
Hospital 17	ℒ (41162, 45495)	ℒ (5743, 6348)	ℒ (2775, 3067)
Hospital 18	ℒ (37216, 41134)	ℒ (2704, 2988)	ℒ (1869, 2065)
Hospital 19	ℒ (34032, 37615)	ℒ (3921, 4334)	ℒ (1704, 1884)
Hospital 20	ℒ (8846, 9778)	ℒ (1482, 1638)	ℒ (689, 761)

Abbreviations: NOOU: number of outpatients; NOIN: number of inpatients; NOSU: number of surgeries.

**Table 4:** The extent of inefficiency for 20 Iranian public hospitals.

Hospitals	Belief Degree				
	0%	25%	50%	75%	100%
Hospital 01	13.43488	13.75816	14.10025	14.48392	14.87131
Hospital 02	0.00000	0.00000	0.00000	0.00000	0.00000
Hospital 03	10.61700	10.89819	11.19201	11.51168	11.83541
Hospital 04	8.22185	8.45835	8.67954	8.86653	9.04879
Hospital 05	4.83058	4.95892	5.10777	5.29900	5.49741
Hospital 06	6.05503	6.22125	6.39708	6.59224	6.79144
Hospital 07	8.94735	9.17307	9.41139	9.67641	9.94443
Hospital 08	0.51054	0.50404	0.49700	0.49061	0.48225
Hospital 09	3.67848	3.78079	3.89674	4.04001	4.18773
Hospital 10	0.03883	0.01603	0.00000	0.00000	0.00000
Hospital 11	0.00000	0.00000	0.01237	0.05015	0.09178
Hospital 12	2.40902	2.47299	2.54859	2.64826	2.75178
Hospital 13	1.97477	1.99968	2.03026	2.07488	2.11884
Hospital 14	4.04720	4.14449	4.24171	4.33951	4.43630
Hospital 15	0.00000	0.00000	0.00000	0.00000	0.00000
Hospital 16	10.57316	10.83996	11.11992	11.42801	11.73908
Hospital 17	16.27186	16.70051	17.15668	17.66985	18.19250
Hospital 18	11.34755	11.63266	11.92967	12.25247	12.57793
Hospital 19	13.86641	14.19261	14.53618	14.91889	15.30372
Hospital 20	8.37461	8.58316	8.80013	9.03519	9.27134
Objective	125.19910	128.33490	131.65730	135.37760	139.14200

**Table 5:** The extent of efficiency for 20 Iranian public hospitals.

Hospitals	Belief Degree				
	0%	25%	50%	75%	100%
Hospital 01	0.29998	0.30044	0.30059	0.30014	0.29966
Hospital 02	1.00000	1.00000	1.00000	1.00000	1.00000
Hospital 03	0.41630	0.41626	0.41595	0.41510	0.41422
Hospital 04	0.63343	0.63287	0.63276	0.63354	0.63441
Hospital 05	0.73757	0.73752	0.73669	0.73435	0.73187
Hospital 06	0.44282	0.44254	0.44191	0.44059	0.43919
Hospital 07	0.46695	0.46721	0.46713	0.46636	0.46556
Hospital 08	0.94855	0.95041	0.95224	0.95392	0.95570
Hospital 09	0.73956	0.73928	0.73834	0.73613	0.73383
Hospital 10	0.99340	0.99733	1.00000	1.00000	1.00000
Hospital 11	1.00000	1.00000	0.99844	0.99387	0.98911
Hospital 12	0.79549	0.79545	0.79467	0.79243	0.79008
Hospital 13	0.69256	0.69541	0.69756	0.69818	0.69884
Hospital 14	0.76974	0.77014	0.77052	0.77085	0.77121
Hospital 15	1.00000	1.00000	1.00000	1.00000	1.00000
Hospital 16	0.27432	0.27453	0.27449	0.27397	0.27342
Hospital 17	0.23390	0.23390	0.23360	0.23275	0.23185
Hospital 18	0.26915	0.26937	0.26939	0.26900	0.26858
Hospital 19	0.22239	0.22286	0.22311	0.22289	0.22265
Hospital 20	0.11626	0.11640	0.11644	0.11627	0.11609

## Data Availability

The data used in this study are available from the authors and can be shared upon reasonable request.
